# Effect of Environmental Variables on African Penguin Vocal Activity: Implications for Acoustic Censusing

**DOI:** 10.3390/biology12091191

**Published:** 2023-08-31

**Authors:** Franziska Hacker, Francesca Terranova, Gavin Sean Petersen, Emma Tourtigues, Olivier Friard, Marco Gamba, Katrin Ludynia, Tess Gridley, Lorien Pichegru, Nicolas Mathevon, David Reby, Livio Favaro

**Affiliations:** 1 ENES Bioacoustics Research Team, University of Saint-Etienne, 42100 Saint-Etienne, France; emma.tourtigues@gmail.com (E.T.); mathevon@univ-st-etienne.fr (N.M.); dreby@me.com (D.R.); 2 Department of Life Sciences and Systems Biology, University of Turin, 10124 Turin, Italy; francesca.terranova@unito.it (F.T.); olivier.friard@unito.it (O.F.); marco.gamba@unito.it (M.G.); 3 Southern African Foundation for the Conservation of Coastal Birds (SANCCOB), Cape Town 7441, South Africa; gavin@sanccob.co.za (G.S.P.); katta@sanccob.co.za (K.L.); 4 Department of Biodiversity and Conservation Biology, University of the Western Cape, Robert Sobukwe Road, Bellville 7535, South Africa; 5 Statistics in Ecology, Environment and Conservation, Department of Statistical Sciences, University of Cape Town, Rondebosch, Cape Town 7701, South Africa; nam.dolphin.project@gmail.com; 6 Institute for Coastal and Marine Research, Nelson Mandela Metropolitan University, Port Elisabeth 6031, South Africa; lorien.pichegru@mandela.ac.za; 7 Institut Universitaire de France, Ministry of Higher Education, Research and Innovation, 1 rue Descartes, CEDEX 05, 75231 Paris, France; 8 CAPE Department, Stazione Zoologica Anton Dohrn, 80121 Naples, Italy

**Keywords:** passive acoustic monitoring, remote monitoring, remote census, *Spheniscus demersus*, vocalisations

## Abstract

**Simple Summary:**

Most seabird species are in need of effective conservation, with 43% being near to globally threatened. Passive acoustic monitoring could serve as a cost-effective, noninvasive population monitoring tool essential for informing future conservation efforts. As such, we set out to investigate whether passive acoustic monitoring could successfully predict the African penguin density at a remote colony in Betty’s Bay, South Africa. We first automated the detection and counting of penguins’ vocalisations in our recordings to facilitate the handling of large datasets. Then, we investigated whether temperature, humidity, and wind speed affected the calling rate of penguins, which would be essential for an accurate census. Finally, taking into account the variations with weather conditions, we showed that passive acoustic monitoring could successfully predict the number of callers within a 10.5 m radius around our devices, indicating that it can be used for cost-effective, noninvasive censuses of African penguin colonies.

**Abstract:**

Global biodiversity is in rapid decline, and many seabird species have disproportionally poorer conservation statuses than terrestrial birds. A good understanding of population dynamics is necessary for successful conservation efforts, making noninvasive, cost-effective monitoring tools essential. Here, we set out to investigate whether passive acoustic monitoring (PAM) could be used to estimate the number of animals within a set area of an African penguin (*Spheniscus demersus*) colony in South Africa. We were able to automate the detection of ecstatic display songs (EDSs) in our recordings, thus facilitating the handling of large datasets. This allowed us to show that calling rate increased with wind speed and humidity but decreased with temperature, and to highlight apparent abundance variations between nesting habitat types. We then showed that the number of EDSs in our recordings positively correlated with the number of callers counted during visual observations, indicating that the density could be estimated based on calling rate. Our observations suggest that increasing temperatures may adversely impact penguin calling behaviour, with potential negative consequences for population dynamics, suggesting the importance of effective conservation measures. Crucially, this study shows that PAM could be successfully used to monitor this endangered species’ populations with minimal disturbance.

## 1. Introduction

Global biodiversity is in rapid decline [1,2], which is evident across a range of habitats, including agricultural lands [3], freshwater ecosystems [4], the Arctic [5], and Antarctica [6,7], but especially apparent in marine and coastal regions [8,9]. Seabirds, making up around 3.5% of all avian species, have been classified as the most threatened avian group, characterised by significantly poorer conservation statuses than other birds [10]. Population declines over the last 20 years have been recorded in approximately half of all seabird species, and those of most concern are penguins (Sphenisciformes) and albatrosses and petrels (Procellariiformes), together representing around 43% of all seabird species [10]. Since many seabird species are critical indicators for the health of their respective marine ecosystems [11,12], their continued decline has drastic implications for not only their conservation but also the broader status of our oceans and coastal habitats. This is supported by the fact that key threats to seabirds are anthropogenic activities known to negatively affect marine ecosystems on larger scales, such as overfishing [13], pollution [14], habitat degradation [15], incidental mortality after by-catch in fisheries gear [16], and human disturbances at colonies, such as tourism [10,17,18].

Focusing on the conservation of umbrella species often allows for protecting the status of their habitat and that of other species living within the same space. Selecting just a single species can be beneficial for the conservation and protection of large areas [19], with studies reporting positive effects on the conservation of intact and restoration of degraded forests [20], and on aquatic biodiversity [21]. Thus, given the importance of seabirds as indicator species, conserving seabird umbrella species could help prevent both the decline of their respective populations and that of their habitat and other marine species within it.

Penguins (Spheniscidae) are a family of seabirds inhabiting most of the southern parts of our planet. Out of the 19 penguin species recognised today [22], two are classified as near threatened, four as vulnerable, and five as endangered [23]. Recently, three penguin species have been internationally voted as highest priority for conservation efforts [24]; one of which, the African penguin (*Spheniscus demersus*), is characterised by a largely depleted population [23], with many island colonies subject to drastic declines and collapses, and only two mainland colonies across South Africa [25,26]. Both individual survival and colony breeding success are strongly impacted by habitat loss owing to resource competition with industrial fisheries, the expansion of anthropogenic activities, and marine noise pollution [26,27,28,29]. Moreover, the negative consequences of oil spills alone may be sufficient to lead to the extinction of the African penguin [27,30,31]. Thus, well-founded conservation actions are urgently needed to counteract their decline and aid populations’ recovery.

The efficacy of such conservation actions must be assessed to ensure they are successful in increasing breeding success or breeding numbers. In that regard, improving our understanding of population trends, threats, life history, distribution, and ecology are priority research areas that can inform and support conservation efforts [10,32]. Considering the African penguin’s sensitivity to human disturbances and the recommendations within the African Penguin Management Plan [33], noninvasive monitoring tools are essential to minimise the adverse effects of further studies of this species’ population trends and breeding ecology on site. Observational studies or monitoring via visual remote sensing tools, such as camera traps or drones, can provide essential insights into colony dynamics and breeding success but often still require human presence in sensitive areas and can be hindered by factors such as the need for good weather conditions and the limited spatiotemporal resolution [34]. Because African penguins rely extensively on acoustic communication for intraspecific communication, a powerful, noninvasive and cost-effective way of monitoring penguin colonies could be passive acoustic monitoring (PAM), which would keep disturbances to a minimum [35] while effectively assessing the number of birds within an area. Specifically, the distinctiveness of the ecstatic display song (EDS), which consists of a sequence of short syllables (type A) followed by a long syllable (type B) and an audible inspiration (type C), and the frequency at which it is produced could make it a good target for detection in recordings [36]. Further, EDSs are important in territory defence and mate choice in African penguins, with especially high calling rates during the beginning of a breeding season [37].

To date, PAM has been used to investigate the relationship between acoustic activity and colony density in a variety of bird species (e.g., eastern wood pewee (*Contopus virens*) [38], Forster’s terns (*Sterna fosteri*) [35], bell miner (*Manorina melanophrys*) [32], short-tailed shearwaters (*Ardenna tenuirostris*) [39], Magellanic (*S. magellanicus*) and southern rockhopper (*Eudyptes chrysocome*) penguins [34]). A review of multiple PAM surveying attempts showed that 79% of studies investigating a relationship between the number of vocalisations and bird density or abundance obtained counts that agreed with those obtained from human surveyors [40]. Additionally, in African penguins, acoustic indices such as the acoustic entropy index (H) have already been shown to be valuable tools for predicting the number of both EDSs and mutual display songs (MDSs) in soundscape recordings of colonies, reflecting overall vocal activity [41]. However, not all studies report the successful application of PAM to estimate population densities [42,43] and some suggest combining them with other survey methods for accurate results [40]. Therefore, further investigations of its applicability for, and accuracy in, the remote monitoring of African penguin colonies are needed to effectively support the development of successful conservation efforts [33].

To investigate whether PAM could be a viable tool to estimate the density within a colony, we deployed a series of static acoustic monitoring devices within the Stony Point penguin colony (Betty’s Bay, South Africa), a key mainland colony with around 1500 breeding pairs as of July 2022 (~7% of the total African penguin population) [41]. To facilitate data handling and provide a modern, time-efficient strategy for counting penguin vocalisations in audio recordings, we first aimed to automate EDS detection. Then, we investigated to what extent weather variables affected calling rate, which can guide the ideal timing of remote acoustic breeding censuses and improve their accuracy. Lastly, taking those variations into account, we estimated the animal density within an area by examining the relationship between total EDS numbers in our recordings and visually observed penguins and callers to trial a novel, noninvasive counting tool useful for this species’ conservation. As such, our study aimed to provide the first steps for the development of a time-efficient remote monitoring tool for the endangered African penguin while simultaneously providing insights into the broader use of PAM for the management and conservation of sensitive, cryptic species.

## 2. Materials and Methods

### 2.1. Acoustic Recordings and Weather Data

**Figure 1:**
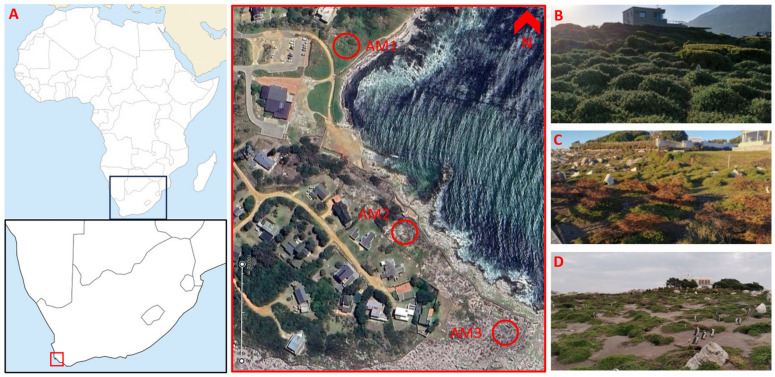
Map and satellite image of the penguin colony and pictures of the three respective recording sites. (**A**) A map of the location of the penguin colony within Africa and South Africa and satellite image of the entire Stony Point penguin colony, with red circles indicating the locations of the acoustic sensors. The righthand panels (**B**–**D**) show pictures of our three recording sites AM1, AM2, and AM3, respectively.

We deployed three acoustic sensors (AudioMoth (AM); Open Acoustic Devices, 2022) in IPX7 waterproof cases (48 kHz sampling rate, no filters applied) throughout the African penguin colony at Stony Point (34°37′14.21″ S, 18°89′32.65″ E) in Betty’s Bay, South Africa, and recorded the colony continuously from March to July 2022. The daily recording schedule, from 4:00–9:00 and 16:30–21:30 South African Standard Time (SAST), was based on previous investigations of peak acoustic activity at the same colony [41] and adjusted to local sunrise and sunset times. This schedule resulted in a total of *n* = 6627 30 min recordings (198,810 total mins) collected over 113 consecutive days. The recorders were placed at locations with slightly different flora, shown on a map in Figure 1. One device was set up in an area of dense dune spinach (*Tetragonia decumbens*) bushes covering the ground completely (AM1), one in a grassy area dispersed with *Baccharis halimifolia* bushes (AM2), and one at the southern end of the colony, where most penguin nests are sand burrows covered by some dune spinach (AM3). AM1 and AM2 were spaced approximately 330 m apart and AM2 was around 130 m from AM3. This layout would allow for an investigation of how vegetation influenced penguin density and calling rate. The acoustic sensors were tied to wooden poles at a height of around 20 cm and oriented to face downwards to minimise directionality that could be introduced by the device casing and batteries. Devices were collected for data download once per week to minimise disturbance.

A 3879 Diastella (March–June) and a Bresser WIFI Colour Weather Station (June and July) set up according to the manufacturer’s instructions measured temperature, humidity, wind direction and speed, barometric pressure, and precipitation rate at the colony through a rain gauge, wind cups and vane, and a thermohygrometer. Additionally, the temperature measured using the AM devices at the beginning of each recording session was extracted using an adapted Python script (Open Acoustic Devices, 2020; Supplementary Information). 

### 2.2. Visual Counting of Penguins

Visual observations were conducted twice throughout the breeding season: in early May and late June/early July, representing the middle and end of one breeding cycle, respectively. Here, the middle of a breeding cycle is defined as the period during which most breeding pairs have chicks fully covered in their juvenile down feathers, whereas the end of the cycle concerns the period during which most chicks have or are about to fledge and leave the nests as “blues” with waterproof plumage. A 10.5 m radius around each AM device, measured with a rangefinder, was marked with wooden poles to allow for the accurate counting of penguins within a set radius. This radius was limited by the layout of the colony, which includes some areas as narrow as 11–12 m between the boardwalk and private property. Across a period of 10 consecutive days, an experimenter (F.H.) counted penguins twice daily, at sunrise and sunset, noting all visible penguins every five minutes and all heard EDS vocalisations and identifying all callers within the radius around the AM device for half an hour per site, resulting in a total of *n* = 48 observation periods. Since visual sexing of African penguins is unreliable [44], both males and females were counted. Observation times were shifted with sunrise and sunset in a way that the first two sites were surveyed in the half hour before and after sunrise, while the third site was surveyed either in the half hour before or after sunset. The schedule was rotated to ensure that every site was surveyed at each of the four possible timeslots (before/after sunrise/sunset) at least twice during each 10-day observation period. Additionally, we counted the number of occupied nests within the radius around the AM devices and calculated the resulting maximum penguin densities. The obtained number of callers within our defined radius and the overall EDS numbers in our recordings would then allow us to investigate whether the number of breeding pairs around the acoustic sensors could be estimated from our recordings. 

### 2.3. Data Analysis

**Figure 2:**
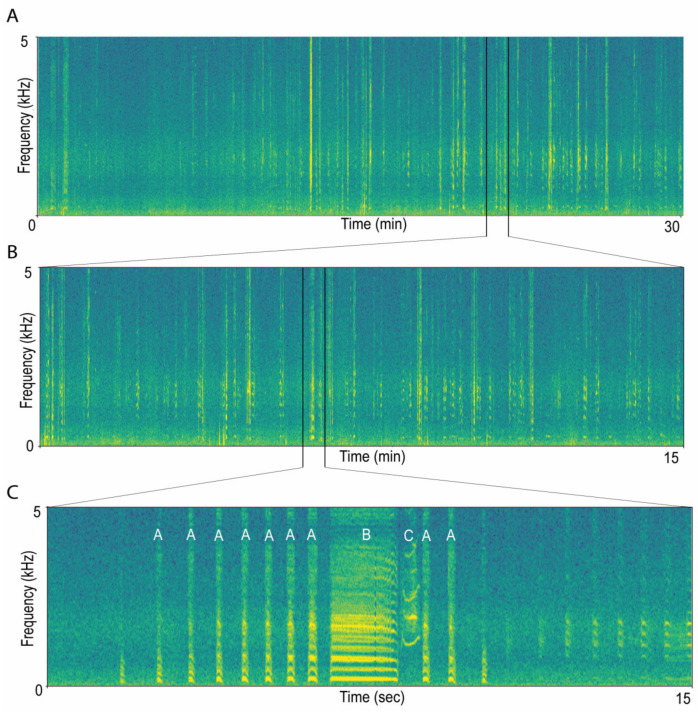
An example recording containing EDS vocalisations. Panel (**A**) shows a 30 min recording from one of our remote acoustic recorders taken at the Stony Point penguin colony, Betty’s Bay, South Africa. Panel (**B**) represents the latter 15 min of the same recording, with single EDSs becoming visible, and panel (**C**) visualises a single, typical EDS vocalisation from the same recording, with A, B, and C indicating the repeated short syllables, the long detection target syllable, and the inspiration syllable, respectively. The figure was created using Raven Pro v. 1.5.0 (Spectrogram window size: 1024, Hann window, overlap 50%).

The collected audio files were uploaded onto the OCCAM SuperComputer [45] at the Competence Centre for Scientific Computing of the University of Turin. To automate the detection of the number of EDSs inside the audio recordings, we used the monitoR [46] package in R v.4.2.3 [47]. A typical EDS is usually composed of many short syllables (A) followed by a long one (B) and an audible inspiration (C) [48], as visualised in Figure 2. The large variations in the number of A syllables in a song [37] and the everchanging characteristics of C syllables (e.g., frequency and frequency modulation, duration, etc.) meant that focussing on the B-syllable for automatic detections would be the most reliable means of acquiring accurate EDS numbers.

As such, using five different B-syllable recordings, binary point-matching templates were created through the monitoR automatic template creation tool [49]. Since different templates could detect the same syllable, we merged all detections of different templates found in the same minute (±1 s). Different threshold scores, above which any detections would be counted as EDSs, were tested (20, 21, 22, 25, 27, and 30) to identify the most accurate one based on visual spectrogram inspections and randomly generated detection curves. Figure S1 in Supplementary Information (SI) visualises one of the five created templates and detection curves of different scores. A threshold of 21 was found to be the most accurate, and as such, detections with scores ≥ 21 were classified as EDSs. To calculate the detector’s average sensitivity (true positive rate), all true positive (TP) and false negative (FN) detections in *n* = 678 (around 10% of our final dataset) audio files were manually counted using spectrographic inspection. Then, the true positive rate was calculated as TP/(TP + FN) and found to be 65.79%. Additionally, the accuracy of automatic detections was crosschecked by two independent observers (F.H. and F.T.) conducting manual spectrographic counts of EDSs with visually assessed good or very good signal-to-noise ratio (SNR) [50] on a subset of audio files (*n* = 199) and validated using Spearman’s rank correlation test.

### 2.4. Statistics–Modelling

A generalised linear mixed model (GLMM) approach was used to investigate the impact of weather conditions on calling rate and the correlation between the average number of identified callers and the number of recorded EDSs using the glmmTMB package [51] in R [47], comparing their respective full and null models. The first model analysed the effects of temperature, wind speed, humidity, and location on the number of EDSs recorded per 30 min while controlling for the recording date and time. Then, we created the second GLMM to investigate whether the number of automatically detected EDSs in our recordings correlated with the average number of penguins and callers counted within the radius around the respective AM device while taking into account call rate variations with weather conditions and controlling for location, time, and date of recording. In both, correlation among all predictors was assessed using variance inflation factor (VIF) analysis and significance was investigated using a chi-square test. Location was controlled for in the second GLMM based on the first model’s results highlighting a significant effect between the recorders. This showed whether PAM could be a reliable census tool for the defined area of detection despite the influences of weather on calling rate. Graphs were made using the sjPlot package [52] in R.

## 3. Results

### 3.1. Automating EDS Counting

We first set out to automate EDS detection in the recordings of our remote acoustic sensors, for which automatic detections were compared with manual spectrographic counts. Spearman’s rank correlation showed a significant correlation between manual and automatic counts, indicating that automatic counts could reflect the relative number of calls present in a 30 min recording (*n* = 199, R = 0.61, *p* < 0.001).

### 3.2. Influence of Environmental Variables on Calling Rate

EDS numbers were significantly correlated with all investigated weather variables and location (full vs. null: x² = 27,836.21, df = 5, *p* < 0.001). Both higher humidity (estimate = 0.003, se = 0.001, *p* < 0.001) and wind speeds (estimate = 0.013, se = 0.001, *p* < 0.001) led to an increase in EDS production. In contrast, higher temperatures led to a decrease in calling rate (estimate = −0.028, se = 0.001, *p* < 0.001). Lastly, AM1 recorded significantly less EDSs than AM2 (estimate = 0.619, se = 0.004, *p* < 0.001), but more than AM3 (estimate = −0.031, se = 0.005, *p* < 0.001). This was confirmed by our visual observations and the count of active nests. AM2, characterised by a mixed vegetation of grass, bushes, and artificial burrows, contained 27 nests and had the highest penguin density (0.156 penguins/m²); AM3, located at the southern end of the colony and containing mostly sand burrows and small dune spinach bushes, had 20 nests in its radius and the lowest density (0.116 penguins/m²); and AM1, which was solely surrounded by dune spinach, had a total of 22 nests and the second lowest density (0.127 penguins/m²). Thus, while the environment found at the colony did not allow for multiple replicates of each vegetation type, the high number of observation sessions supported that habitat type may influence the overall penguin density or calling rate; we therefore controlled for location in the subsequent GLMM.

### 3.3. Relationship between EDS Counts and Penguin Abundance

**Figure 3:**
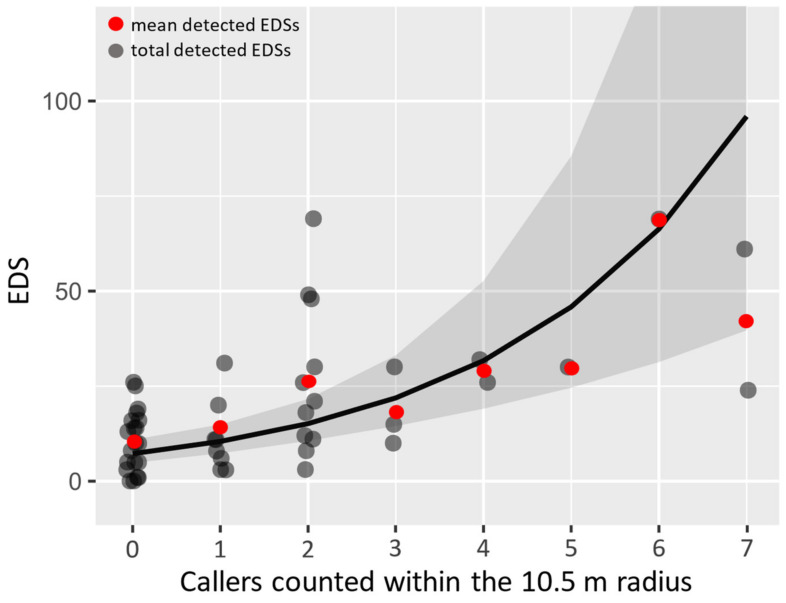
Correlation between the number of visually observed callers and the automatically counted ecstatic display song numbers. An overlay of the model prediction (grey line) along with the 95% confidence interval (shaded grey area). Red dots indicate the mean number of ecstatic display songs (EDS) observed for the respective number of calling individuals within the 10.5 m radius around our recording devices.

Despite the variations in EDS numbers with environmental conditions, our model showed that there was a significant correlation between the number of detected EDSs and the number of callers (the number of penguins seen calling) within the 10.5 m radius (full vs. null: x² = 24.127, df = 2, *p* < 0.001). Specifically, the number of EDSs detected in our recordings was significantly positively correlated with the number of callers visually observed within the 10.5 m radius around our devices (estimate = 0.369, se = 0.075, *p* < 0.001), as shown in Figure 3, but not with the overall average number of penguins present within a radius (estimate = −0.036, se = 0.022, *p* = 0.102), potentially because transiting penguins, those crossing through the radius without having a nest therein, were included in our counts. Thus, future studies should aim to effectively distinguish between resident and transiting penguins to assess whether there is a correlation between resident penguins and EDSs. 

## 4. Discussion

### 4.1. Effectiveness of PAM

Passive acoustic monitoring has been shown to accurately reflect bird population estimates in several species [32,34,35,38,39,40]. Similarly, despite some studies reporting less accurate results [42,43], we revealed here its potential for accurate remote acoustic censuses of an African penguin colony. Despite the identified effects of environmental variables on the number of recorded EDS calls, we showed that there was a positive correlation between the number of EDSs detected in our recordings and the number of callers observed within our radii, indicating that PAM based on EDS detections could be an effective monitoring tool for African penguin colonies.

However, our visual observations showed that the number of active callers within our plots (0–7 penguins) was always lower than the number of active nests within that radius, which lowers the risk of overestimation. Further, within a site, the average number of counted penguins across our observational period (AM1: 7; AM2: 14; AM3: 11) was about half the number of active nests within the site (AM1: 22; AM2: 27; AM3: 20), and even less were identified as active callers (AM1: 0.5; AM2: 0.5; AM3: 2), which often called multiple times within a half-hour survey. However, these low medians and highly variable data may have been caused by the occurrence of days of low vocal activity likely resulting from the fact that our observations took place toward the end of the breeding season. Therefore, to ensure accuracy of a remote censusing tool, we stress the importance of conducting acoustic and visual surveys at the beginning of the breeding season when penguins are most vocally active.

Lastly, the sensitivity of our detector (65.79%) indicates that another counting approach may be needed, since reducing false negatives in monitoR is difficult, with the inclusion of more templates leading to less accurate results.

### 4.2. Impacts on Detection Effectiveness

We further showed that environmental conditions—temperature, wind speed, humidity, and location—affected the detection of EDSs in our recordings, which should be considered when developing this census tool further. Variations between locations and with varying weather conditions may have resulted from changes in vocal activity, penguin density or penguin presence at the colony. Alternatively, differences in sound propagation and detection between our AMs may have played a role. 

First, we showed that all chosen weather variables—temperature, humidity, and wind speed—affected calling rates and should thus be considered on census days, similar to how forecasts are used in planning songbird transect surveys [53,54]. Higher wind speeds may have directly increased the calling rate of penguins by, e.g., lowering the perceived temperature, leading to higher vocal activity, or changed sound propagation in a way that altered song detection at the receiver, our AMs, thus causing variations in overall detection of EDSs at different wind speeds. Alternatively, the increased number of recorded EDSs at higher wind speeds could have resulted from an increased number of false positive detections in our automated detection. However, manual inspections of a portion of the dataset showed that overestimations of EDSs did not always occur on days with higher wind speeds. Similarly, higher humidity may be indicative of rainy days with relatively lower or lower perceived temperatures on which penguins may call longer or more frequently, which could explain the increased occurrence of EDSs. 

Notably, decreasing the calling rate at higher temperatures could have important implications for both mate selection and territorial disputes [36]. First, during hot weather, fewer males may be prone to call, potentially leading to fewer new pairs formed at the beginning of a breeding season, possibly resulting in more nonbreeding adults. Investigations of whether the numbers of nonbreeding adults at colonies during a breeding season are higher after a hotter start to the season may confirm this hypothesis. Second, since vocal contests are usually the first step in territorial conflicts and often serve to assess rivals, they frequently prevent the escalation of fights [55]. Thus, higher temperatures could lead to fewer EDSs produced in territorial defence, which may increase physical territorial fights and injuries, likely negatively affecting penguin survival. Alternatively, the lower number of EDSs could reflect a higher absence of breeding individuals from the colony, spending more time at sea. This could adversely affect chick survival rates, especially in young chicks. As such, the decrease in EDSs at higher temperatures could have negative consequences for the survival rate of African penguins given the current state of our climate and the rising global temperatures [56]. Increased temperatures have already been shown to lead to a shift in the timing of the 2022 breeding season and a decrease in egg survival rate, most recently affected by a mass-abandonment following a hot spell in January 2022 at the Stony Point colony, as indicated by the colony’s research manager (Van Eeden, L., Pers. Comm.). Therefore, effective conservation strategies for the African penguin are necessary.

Furthermore, the variation in detected EDSs across locations suggest that penguin density varies between locations of differing vegetation types, with some areas of the colony supporting a higher local density than others. Alternatively, differences in EDS numbers across locations may be reflective of variations in sound propagation in areas with different vegetation types or changes in calling rates rather than penguin density. However, given that the number of identified active nests and detected EDSs was highest at AM2, the location with the comparatively densest vegetation, it is suggested that habitat suitability is the likely explanation for the observed variations in density rather than changes in calling rate or sound propagation. Notably, despite habitat variations, PAM was successful in identifying the relationship between EDSs and present callers, highlighting its usefulness for the monitoring of species with cryptic nesting behaviour. Furthermore, our results can provide information on suitable vegetation that can support larger numbers of penguins, which can be important for improving existing colonies through habitat rehabilitation actions and the planning of the establishment of artificial colonies [57], which is especially essential given the expansion of urban areas into nature, decreasing the area available to breeding colonies.

### 4.3. Improving Detections for Successful PAM

Others have suggested that an improved understanding of the focal species’ vocal behaviour significantly improves the development of acoustic detections and passive acoustic surveys [58]. Accuracy can be further improved by investigating the following important factors: the ideal census time, specifically distinguishing between breeding and nonbreeding seasons, the female-to-male singing ratio, and an expansion of the target vocalisations and sampling radius. 

Since previous research has suggested that EDSs have functions of both territory defence and mate attraction [48], differences in the overall number of produced EDSs are likely to appear between different stages of the breeding season. Thus, future investigations should attempt to compare accuracy across all stages, such as the beginning, middle, and end, to identify the ideal time of year or breeding season period during which the number of EDSs most accurately reflects the number of active breeding pairs.

Furthermore, previous research on the female-to-male singing ratio reports that females produce EDSs, especially in territory defence [59], but further investigations of this ratio are necessary to accurately estimate breeding pairs. On one hand, since visual sexing is not possible in African penguins, assuming all callers to be a male may lead to an overestimation of breeding pairs. On the other hand, depending on the female-to-male singing ratio and the census timing, the fact that only one parent is usually present at a nest during the chick-rearing period and both sexes produce EDSs may be beneficial for acoustic censuses, since breeding pairs could still be counted correctly.

Additionally, the detection radius may be increased to improve the overall detection of African penguins further. The fact that only a small portion of EDSs came from callers within our radii may have resulted from including a relatively narrow radius compared with the detection range of our AM devices. Similarly, since not all present penguins were observed to produce EDSs, sampling could be expanded to include a larger portion of the African penguin vocal repertoire, which consists of six identified calls and songs [36]. This suggestion is supported by recent efforts to estimate population density in Magellanic and southern rockhopper penguins using PAM, which included sounds other than calls, such as huffs and sneezes, and obtained an accurate estimate [34].

Lastly, some have suggested that PAM is most efficient when combined with other noninvasive census strategies [40], such as thermal sensing. The usefulness of thermal sensing has recently been investigated in a study of American woodcock (*Scolopax minor*) detection probability [60], which pointed out that, in forests, its accuracy declined with vegetation density. However, given the absence of a forest-like environment with dense trees found at our study sites, thermal sensing may be useful to support remote acoustic censuses. This will be especially important for establishing whether the absence of EDSs in recordings correlates to penguin absence or silence.

## 5. Conclusions

We showed that PAM has the potential to become a useful, low-cost monitoring tool for sensitive seabird species such as the African penguin despite the influences of environmental variables on the number of detected EDSs in AM recordings. The detection of African penguin EDS vocalisations was successfully automated, as confirmed by a comparison between manually counted and automatically detected EDSs in our recordings. We also found that humidity, wind speed, and temperature significantly affected EDS production, with an increase at higher humidity and wind speeds and a decrease at higher temperatures. Lastly, taking these weather effects into account, we showed a significant correlation between the number of detected EDSs and the number of callers within a 10.5 m radius around our AM devices.

To further improve the accuracy of remote PAM censuses of this species and their efficiency, the above outlined limitations should be considered in the further development of this tool. Specifically, future research aiming to improve it further should focus on determining the ideal census time within a breeding season, accurate assessment of the female-to-male singing ratio to more accurately predict breeding pair numbers based on EDS vocalisations, and an expansion of the target vocalisations and sampling radius.

## Data Availability

The data presented in this study are available upon request from the corresponding author. The data are not publicly available, as recordings may contain voices of staff and visitors of the colony.

## Supplementary Materials

The following supporting information can be downloaded at: https://www.mdpi.com/2079-7737/12/9/1191/s1. The adapted Python script for AudioMoth parameter extraction; Figure S1: Example of B-syllable recording and corresponding monitoR template; Figure S2: Example of a detected EDS in an AudioMoth recording.
