# Supplementary material for: Effect of Environmental Variables on African Penguin Vocal Activity: Implications for Acoustic Censusing
Source: Biology (Basel). 2023 Aug 31;12(9):1191. doi: 10.3390/biology12091191 (PMC10525562; doi:10.3390/biology12091191)
Supplement: Supplementary file 1 [file biology-12-01191-s001.zip › biology-2523572-supplementary.pdf]

biology

MDPI

Supplementary Material for: Effect of Environmental Variables on African Penguin Vocal Activity: Implications for Acoustic Censusing

Franziska Feist [1,*], Francesca Terranova [2], Gavin Sean Petersen [3], Emma Tourtigues [1], Olivier Friard [2], Marco Gamba [2], Katrin Ludynia [3,4], Tess Gridley [5], Lorien Pichegru [6], Nicolas Mathevon [1,7], David Reby [1,7,†] and Livio Favaro [2,8,*,†]

1 ENES Bioacoustics Research Team, University of Saint-Etienne, 42100 Saint-Etienne, France; emma.tourtigues@gmail.com (E.T.); mathevon@univ-st-etienne.fr (N.M.); dreby@me.com (D.R.)
2 Department of Life Sciences and Systems Biology, University of Turin, 10124 Turin, Italy; francesca.terranova@unito.it (F.T.); olivier.friard@unito.it (O.F.); marco.gamba@unito.it (M.G.);
3 Southern African Foundation for the Conservation of Coastal Birds (SANCCOB), Cape Town 7441, South Africa; gavin@sanccob.co.za (G.S.P.); katta@sanccob.co.za (K.L.)
4 Department of Biodiversity and Conservation Biology, University of the Western Cape, Robert Sobukwe Road, Bellville 7535, South Africa
5 Statistics in Ecology, Environment and Conservation, Department of Statistical Sciences, University of Cape Town, Rondebosch, Cape Town 7701, South Africa; nam.dolphin.project@gmail.com
6 Institute for Coastal and Marine Research, Nelson Mandela Metropolitan University, Port Elisabeth 6031, South Africa; lorien.pichegru@mandela.ac.za
7 Institut Universitaire de France, Ministry of Higher Education, Research and Innovation, 1 rue Descartes, 75231 Paris Cedex 05, France
8 CAPE Department, Stazione Zoologica Anton Dohrn, 80121 Naples, Italy
* Correspondence: ffeist98@gmail.com (F.F.); livio.favaro@unito.it (L.F.)
† Co-senior authors.

1. Adapted Python script for AudioMoth parameter extraction

```
import sys
import re
import csv
from os import listdir
from os.path import isfile, join
from datetime import datetime, timezone, timedelta

directory = sys.argv[1]
COMMENT_START = 0x38
COMMENT_LENGTH = 0x180
files = [f for f in listdir(directory) if isfile(join(directory, f)) and ".WAV" in f.upper()]
with open("comments.csv", "w", newline="") as csvfile:
    csvWriter = csv.writer(csvfile, delimiter=",")
    csvWriter.writerow(["Index", "File", "Time", "Battery (V)", "Temperature (C)",
"Comment"])
    for i, fi in enumerate(sorted(files)):
        print(fi)
        with open(join(directory, fi), "rb") as f:
            # Read the comment out of the input file
            f.seek(COMMENT_START)
            comment = f.read(COMMENT_LENGTH).decode("ascii").rstrip("\0")
            # comment = ""
            # Read the time and timezone from the header
```

```
ts = re.search(r"(\d\d:\d\d:\d\d \d\d/\d\d/\d\d\d\d)", comment)[1]
tz = re.search(r"\(UTC([-|+]\d+)?:?(\d\d)?\)", comment)
hrs = 0 if tz[1] is None else int(tz[1])
mins = 0 if tz[2] is None else -int(tz[2]) if hrs < 0 else int(tz[2])
timestamp = datetime.strptime(ts, "%H:%M:%S %d/%m/%Y")
timestamp = timestamp.replace(tzinfo=timezone(timedelta(hours=hrs, minutes=mins)))
# Read the battery voltage and temperature from the header
battery = re.search(r"(\d\.\d)V", comment)[1]
temperature = re.search(r"(-?\d+\.\d)C", comment)[1]
# Print the output row
csvWriter.writerow([i, fi, timestamp.isoformat(), battery, temperature, comment])
```

2. Figure S1: Example of a B syllable recording and the corresponding monitoR template

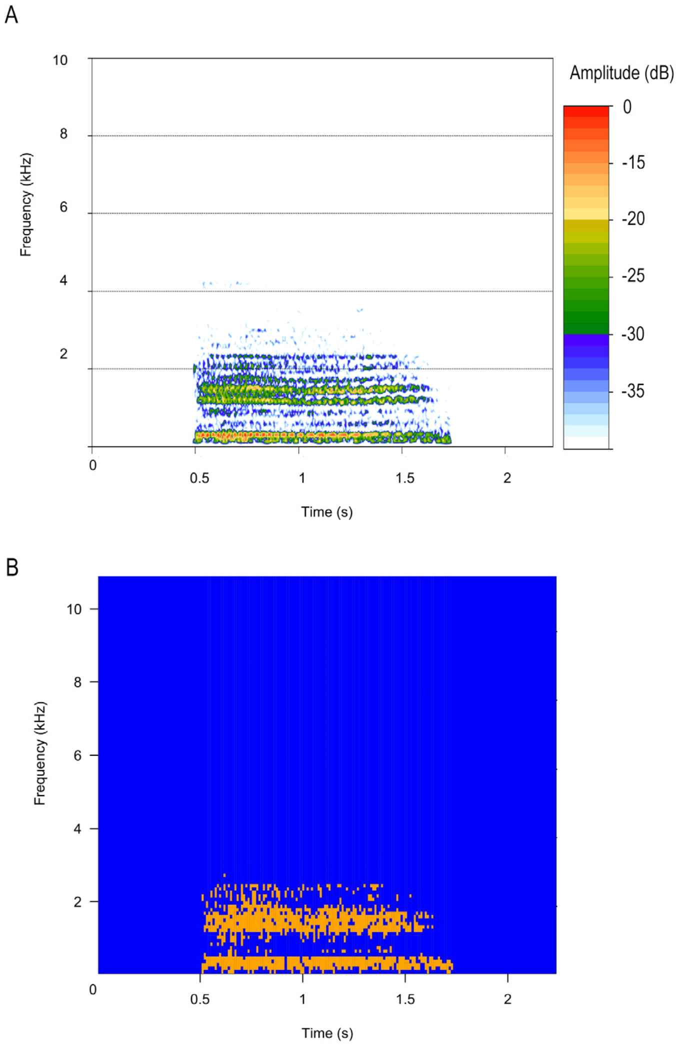

Figure S1. One of five B syllable recordings and the binary point detection template created from it. (A) shows the spectrogram of one of our five B syllable recordings used for the creation of our

detection templates. (B) visualises the binary point detection template created from the same recording using the automatic template creation tool of monitor.

3. Figure S2: Example of a detected EDS in an AudioMoth recording

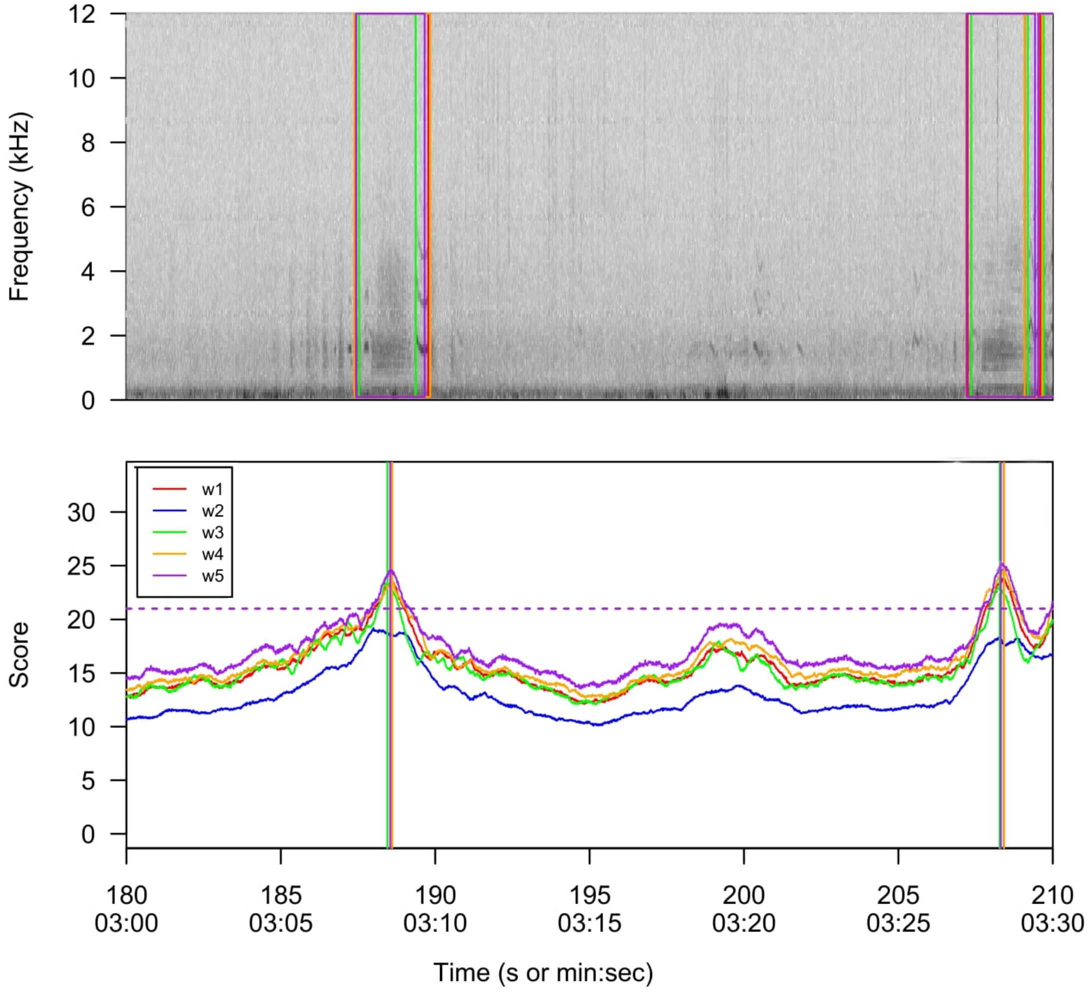

Figure S2. Example detection of two Ecstatic Display Songs (EDS) in one of our recordings. The top panel shows the spectrogram with two visible EDS, while the bottom panel shows the corresponding detection curves of all five detection templates (w1–w5). The horizontal, purple-dotted line indicates the selected detection threshold of 21 above which a detection was scored as an EDS.
